# Improved mitochondrial stress response in long‐lived Snell dwarf mice

**DOI:** 10.1111/acel.13030

**Published:** 2019-08-18

**Authors:** Ulas Ozkurede, Richard A. Miller

**Affiliations:** ^1^ Department of Molecular and Cellular Pathology, University of Michigan Geriatrics Center University of Michigan School of Medicine Ann Arbor MI USA; ^2^ Department of Pathology, University of Michigan Geriatrics Center University of Michigan School of Medicine Ann Arbor MI USA

**Keywords:** aging, lifespan extension, mitochondrial stress response, mtUPR, Snell mice

## Abstract

Prolonged lifespan and improved health in late adulthood can be achieved by partial inhibition of mitochondrial proteins in yeast, worms, fruit flies, and mice. Upregulation of the mitochondrial unfolded protein response (mtUPR) has been proposed as a common pathway in lifespan extension induced by mitochondrial defects. However, it is not known whether mtUPR is elevated in long‐lived mouse models. Here, we report that Snell dwarf mice, which show 30%–40% lifespan extension and prolonged healthspan, exhibit augmented mitochondrial stress responses. Cultured cells from Snell mice show elevated levels of the mitochondrial chaperone HSP60 and mitochondrial protease LONP1, two components of the mtUPR. In response to mitochondrial stress, the increase in *Tfam* (mitochondrial transcription factor A), a regulator of mitochondrial transcription, is higher in Snell cells, while *Pgc‐1α*, the main regulator of mitochondrial biogenesis, is upregulated only in Snell cells. Consistent with these differences, Snell cells maintain oxidative respiration rate, ATP content, and expression of mitochondrial‐DNA‐encoded genes after exposure to doxycycline stress. In vivo, compared to normal mice, Snell mice show stronger hepatic mtUPR induction and maintain mitochondrial protein stoichiometry after mitochondrial stress exposure. Overall, our work demonstrates that a long‐lived mouse model exhibits improved mitochondrial stress response, and provides a rationale for future mouse lifespan studies involving compounds that induce mtUPR. Further research on mitochondrial homeostasis in long‐lived mice may facilitate development of interventions that blunt mitochondrial deterioration in neurodegenerative diseases such as Alzheimer's and Parkinson's and postpone diseases of aging in humans.

## 1 INTRODUCTION

Mouse lifespan can be extended both by genetic alterations and by postnatal treatments (Flurkey, Astle, & Harrison, 2010; Miller et al., 2005; Sun, Sadighi Akha, Miller, & Harper, 2009) starting as late as 75% of the median lifespan of control mice (Harrison et al., 2009). Snell dwarf mice (hereafter “Snell mice”), one of the most studied long‐lived mouse models, have a single point mutation in *Pit1* and show more than 40% increase in mean and maximal lifespan (Flurkey, Papaconstantinou, Miller, & Harrison, 2001). Snell mice exhibit abnormalities in pituitary gland development and in turn have reduced growth hormone (GH) production (Sinha, Salocks, & Vanderlaan, 1975) and decreased levels of circulating insulin‐like growth factor 1 (IGF1) (van Buul‐Offers et al., 1994). The alterations in GH pathway seem to be important for longevity, since growth hormone receptor knockout (GHR‐KO), GH‐releasing‐hormone knockout (GHRH‐KO) mice, and *Ghrhr^lit/lit^* mice, all of which have disrupted GH signaling, also live longer (Coschigano et al., 2003; Flurkey et al., 2001; Sun et al., 2013). Although the specific mutations resulting in lifespan extension in Snell dwarf mice and in other long‐lived mutant mice are well described with their immediate effects on GH/IGF1 signaling, how these alterations result in prolonged lifespan remains elusive. Modifications in a variety of cellular pathways have been proposed to play a causative role in longevity of these GH/IGF1‐deficient mice, including diminished mTOR signaling (Dominick et al., 2015), increased autophagy (Wang & Miller, 2012), and elevated proteasome activity (Drake et al., 2015; Pickering, Lehr, & Miller, 2015).

Mitochondria‐mediated lifespan extension in *Caenorhabditis elegans* can be induced by a diversity of factors, including mutations in genes encoding electron transport chain (ETC) complexes (Feng, Bussiere, & Hekimi, 2001), RNAi‐mediated knockdown of mitochondrial genes (Hamilton et al., 2005), and pharmacologic treatments inhibiting mitochondrial translation (Tsang, Sayles, Grad, Pilgrim, & Lemire, 2001). Upregulation of the mitochondrial stress response, also called “mitochondrial unfolded protein response (mtUPR),” has been proposed as the common longevity mechanism linking mitochondrial disruption to longevity (Houtkooper et al., 2013). Mitochondrial stress applied early in life by temporary silencing of cytochrome c oxidase‐1 subunit Vb/COX4 (*cco‐1*), which results in lifespan extension in *C. elegans*, also causes mtUPR (*hsp6*) upregulation that persists throughout the lifespan even when the initial stress is withdrawn (Durieux, Wolff, & Dillin, 2011). This observation suggests that interim mitochondrial stress can induce permanent reprogramming of the mtUPR pathway, and it may be the upregulation of mtUPR that leads to lifespan extension. Supporting this idea, transgenic worms with muscle‐specific overexpression of nematode mtUPR protein Hsp70F live more than 40% longer, implying that mtUPR upregulation may be sufficient for lifespan extension in worms (Yokoyama et al., 2002).

Partial defects in mitochondrial genes prolong lifespan not only in worms (Ryan & Hoogenraad, 2007; Wong, Boutis, & Hekimi, 1995) and in flies (Copeland et al., 2009), but also in mice (Liu et al., 2005). *Mclk1* is the mouse orthologue of the *C. elegans* gene *clk‐1*, a mitochondrial enzyme involved in ubiquinone biosynthesis. Although the homozygous deletion of *Mclk1* in mice is lethal (Levavasseur et al., 2001), mice heterozygous for *Mclk1* show prolonged average and maximum lifespan, suggesting evolutionary conservation of this longevity pathway. It is likely that decreased expression of *Mclk1* induces mtUPR, since *Mclk1^+/−^* mice show reduced mitochondrial electron transport and ATP synthesis, indicating mitochondrial defects (Lapointe & Hekimi, 2008). No data, however, are available on the levels of mtUPR in Mclk1+/− mice. Mice with deleted *Surf1*, a putative ETC complex IV gene, also live longer (Dell'agnello et al., 2007), although it is not yet clear if this effect is robust; another study found no significant lifespan extension in female *Surf1^−/−^* mice (Deepa et al., 2018). Recently, it has been reported that primary cells derived from *Surf1^−/−^* mice have increased mtUPR (Pharaoh, Pulliam, Hill, Sataranatarajan, & Van Remmen, 2016), further supporting the idea that upregulation of mtUPR may be required for mouse lifespan extension induced by mitochondrial defects.

First described in mammalian cells, mtUPR is characterized by the upregulation of mitochondrial chaperones HSP60 and HSP10 that is not accompanied by an increase in the expression of chaperones located in the cytosol or endoplasmic reticulum (Martinus et al., 1996). In the absence of mitochondrial stress, HSP60 is required for proper folding of nuclear‐DNA‐encoded mitochondrial proteins imported from the cytosol into the matrix. In addition, HSP60 facilitates refolding of structurally unstable proteins prone to aggregation as part of the mitochondrial stress response. Some misfolded proteins, on the other hand, are degraded and recycled by mitochondrial protease LONP1, which is also upregulated in response to mitochondrial stress (Zhao et al., 2002). A cross‐species study found a correlation between maximum lifespan and HSP60 levels in the liver, heart, and brain among species of mammals (Salway, Gallagher, Page, & Stuart, 2011). However, it is not yet known whether mtUPR is elevated in any of the long‐lived mouse models. We suspected that long‐lived Snell mice, whose longevity reflects pathways not obviously linked to mitochondrial maintenance or biogenesis, might have improved mtUPR. Specifically, we assessed whether Snell mice have higher basal mtUPR activity, show elevated response to mitochondrial stress, and are resistant to effects of mitochondrial stress exposure both at the cellular level and in vivo.

## 2 RESULTS

### 2.1 Snell cells show elevated mtUPR despite comparable mitochondrial abundance

**Figure 1:**
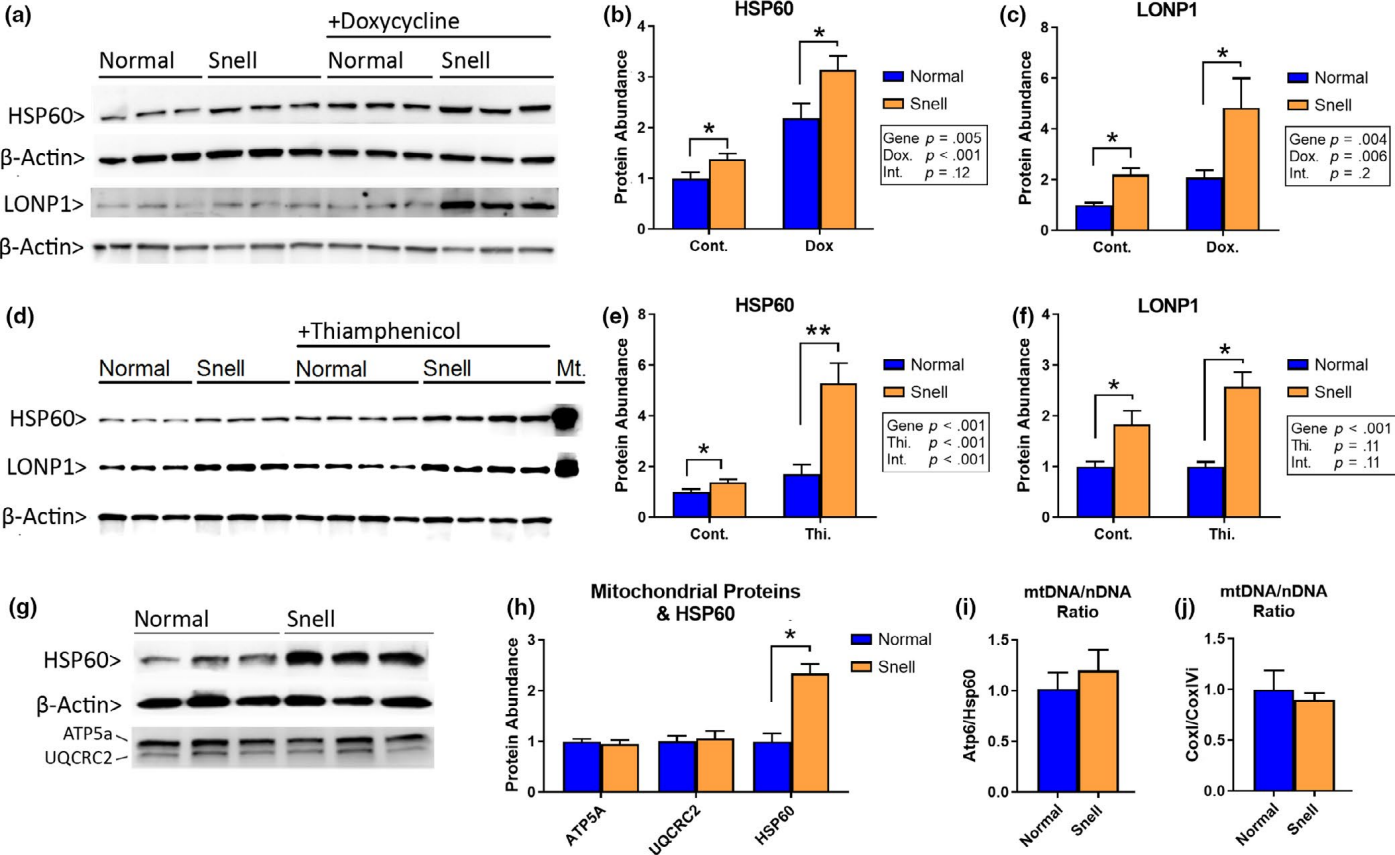
Elevated HSP60 and LONP1 levels in Snell fibroblasts. (a) Immunoblot for HSP60 and LONP1 in normal and Snell cells after 48‐hr exposure to doxycycline or control media. Three mice are shown for each condition. β‐Actin is shown as a loading control. (b) Mean and *SEM* for *N* = 6 mice for HSP60 and (c) LONP1. * indicates *p* < .05; ** indicates *p* < .01 by Student's *t* test for comparison between normal and Snell cells. Results of two‐factor ANOVA are shown in the boxed inset, with *p*‐values for the interaction (“Int.”) effect in this and all subsequent panels. (d) Representative immunoblot for HSP60 and LONP1 in normal and Snell cells after exposure to control or thiamphenicol medium. Column “Mt.” is mitochondria sample isolated from rat heart as positive control. (e) Statistical results for *N* = 3–4 mice in each condition for HSP60 and (f) LONP1. (g) Levels of HSP60 and mitochondrial proteins ATP5a and UQCRC2 in normal and Snell fibroblasts, with actin shown as a control. Three mice of each genotype are shown. (h) Statistical results for *N* = 3 mice of each genotype. (i) Ratio of mitochondrial DNA to nuclear DNA measured as Atp6/Hsp60, and (j) as CoxI/CoxIVi for *N* = 6 mice

HSP60 facilitates folding of mitochondrial peptides into functional proteins, and its expression increases as a part of the mtUPR (Martinus et al., 1996). We compared levels of HSP60 in Snell and control fibroblasts with or without exposure to doxycycline (Dox), an inducer of mtUPR stress. HSP60 levels in Snell cells were significantly elevated compared to normal cells without exposure to Dox (Figure 1a,b). Dox increased HSP60 levels in both normal and Snell cells, as expected (*p* = .001 for drug effect), and Snell cells had higher levels of HSP60 than normal cells, but there was no evidence that the response to Dox differed between the two genotypes (i.e., the interaction term in the two‐way ANOVA was 0.19, not significant). Another indicator of mtUPR, mitochondrial protease LONP1, was also elevated in Snell cells with or without Dox exposure (Figure 1a,c). The degree of LONP1 upregulation in response to Dox did not differ between two cell types (as indicated by the nonsignificant interaction term in the two‐way ANOVA).

We also evaluated responses to thiamphenicol, another inducer of mtUPR, which blocks mitochondrial protein synthesis by interacting with the 28S subunit of the mitochondrial ribosome, a mode of action distinct from that of Dox. We again noted significantly higher levels of HSP60 in Snell cells (Figure 1d,e), regardless of thiamphenicol treatment, but ANOVA showed that the response to this drug was higher in Snell than in control cells (*p* = .0005 for the interaction term). Snell cells had higher LONP1 levels with or without thiamphenicol exposure, but unlike Dox, thiamphenicol had no significant effect on LONP1 levels (Figure 1d,f).

We considered the hypothesis that elevated levels of HSP60 and LONP1 might reflect higher levels of mitochondria in Snell fibroblasts, despite flow cytometric data to the contrary (Page et al., 2009). We therefore measured levels of ATP5a and UQCRC2, mitochondrial ETC proteins encoded in the nucleus, and saw no difference between Snell and control cells (Figure 1g,h). Similarly, we found no differences in the ratio of mitochondrial DNA to nuclear DNA using two different sets of primers (Figure 1i,j), suggesting comparable mitochondrial abundance in two cell types.

Taken together, our results indicate that mitochondrial chaperone HSP60 and mitochondrial protease LONP1, two components of the mitochondrial stress response, are elevated in Snell cells and remain so after exposure to either of the two inducers of mitochondrial stress.

### 2.2 Snell cells are protected from mitochondrial stress

**Figure 2:**
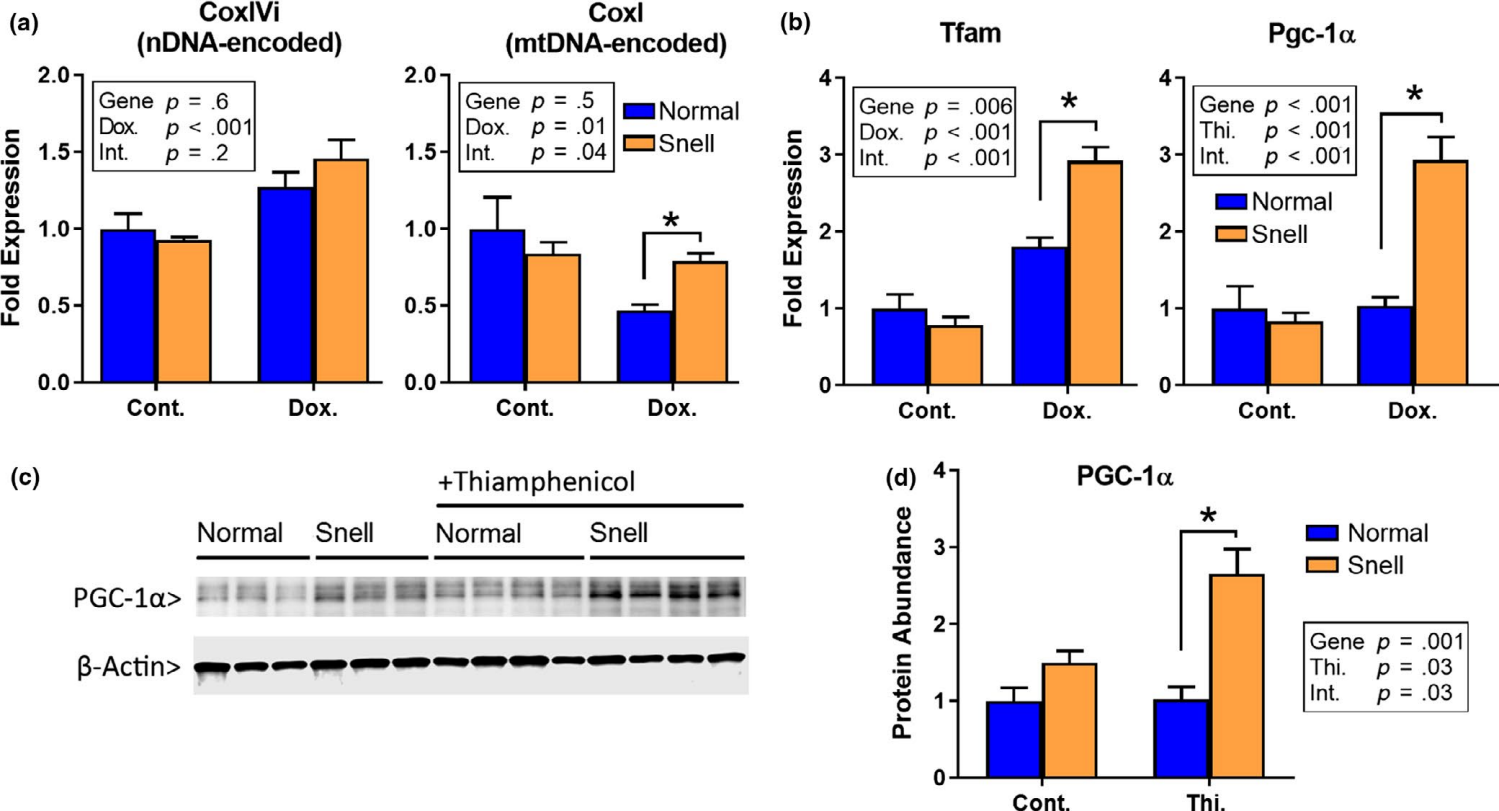
Improved mitochondrial stress response in Snell cells that involves upregulation of Tfam and Pgc1a. (a) Nuclear‐DNA‐encoded (CoxIVi) and mitochondrial‐DNA‐encoded (CoxI) transcript levels, and (b) Tfam and Pgc‐1α mRNA levels after doxycycline treatment in normal and Snell primary fibroblasts for *N* = 6 mice. (c) Western blot image and (d) quantification for PGC‐1α protein expression after thiamphenicol treatment in normal and Snell fibroblasts for *N* = 3–4 mice. * indicates *p* < .05 by Student's *t* test for comparison between normal and Snell cells

The ETC, a multicomponent system required for oxidative respiration, is formed by a combination of peptides, some of which are encoded in nuclear DNA (nDNA) and others in mitochondrial DNA (mtDNA). We measured the change in the levels of mtDNA‐encoded cytochrome c oxidase subunit 1 (*Cox1)* and nDNA‐encoded cytochrome c oxidase subunit 4, each of which is a subunit of ETC complex IV (cytochrome c oxidase). Nuclear‐DNA‐encoded gene expression (*CoxIVi)* was not altered by doxycycline in either Snell or control cells (Figure 2a, *CoxIVi* graph). We observed a decrease of more than 50% in the expression of mtDNA‐encoded *Cox1* in normal cells exposed to doxycycline (*p* = .029), but doxycycline seemed to have no effect in Snell cells (two‐way ANOVA, interaction effect; *p* = .04, indicating a difference between Snell and control mice in response to doxycycline; Figure 2a, *CoxI* graph).

We considered two possible explanations for maintained mtDNA‐encoded gene expression in Snell cells. Efficacy of doxycycline might be low on Snell cells; the failure of the drug might be due, for example, to rapid degradation of the drug by Snell fibroblasts. Alternatively, while doxycycline might reduce mitochondrial gene expression effectively, Snell cells may have a mechanism to compensate for this effect by the 48‐hr time point. To address the first idea, we noted that doxycycline treatment resulted in upregulation of both *Hsp60* and *Hsp10*, confirming that this drug is indeed effective on Snell fibroblasts and activates mtUPR as expected (Figure S1). We tested the latter model by monitoring *Cox1* levels at earlier time points. At *t* = 2 hr, we did not detect a significant decrease in *Cox1* mRNA levels upon doxycycline treatment in either Snell or normal cells (Figure S3a). At *t* = 6 hr, in contrast, *Cox1* abundance decreased in both normal and Snell cells (Figure S3b; *p* = .005 for Dox effect and *p* > .9 for interaction effect by 2‐way ANOVA), indicating that doxycycline is effective in both cell types and induces a reduction in *Cox1* transcript levels. Combined with data at *t* = 48 hr, these results support our interpretation that Snell cells also experience a decrease in mtDNA‐encoded *Cox1* levels initially, which induces mtUPR, but they are able to compensate for the effects of deficient mitochondrial gene expression by 48 hr (Figure S3c).

We next focused on pathways which might compensate for the doxycycline effects by increasing mitochondrial transcription and biogenesis in Snell cells. We found that in response to doxycycline treatment, the level of increase in mRNA for *Tfam* (*m*itochondrial *t*ranscription *fa*ctor *A*), the main regulator of mitochondrial transcription, was twofold higher in Snell cells (Figure 2b). In addition, mRNA for *Pgc‐1α*, the main regulator of mitochondrial biogenesis, was upregulated only in Snell cells (Figure 2b). Similarly, thiamphenicol exposure led to higher levels of PCG‐1α protein in Snell cells compared to control cells (Figure 2c,d).

### 2.3 Snell cells are resistant to mitochondrial stress

**Figure 3:**
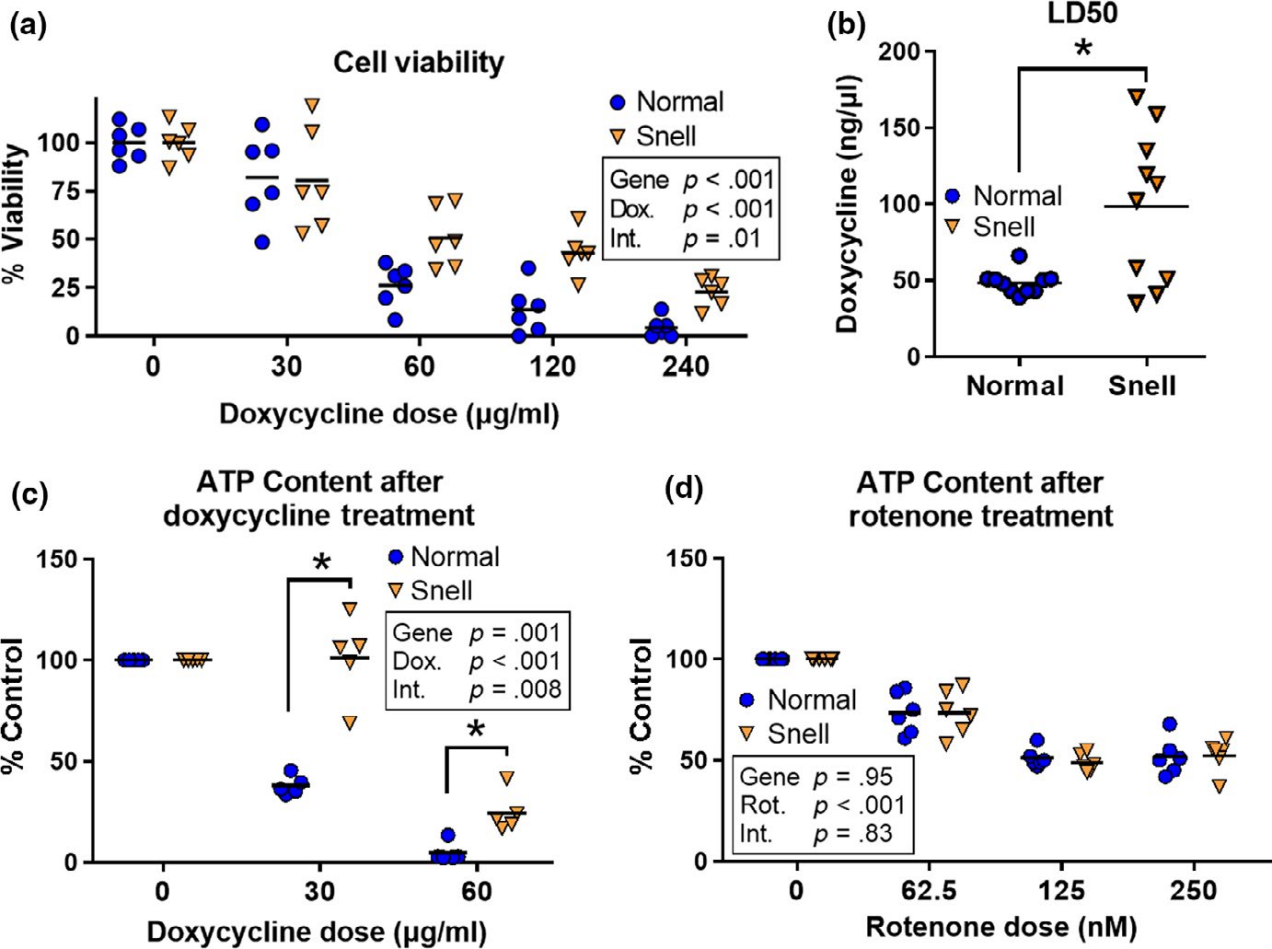
Snell cells are resistant to mitochondrial stress. (a) Cell viability (WST‐1 assay) after 72‐hr incubation in media with varying doses of doxycycline. Each symbol represents cells from an individual mouse; the same mice were used to generate data at each dose of doxycycline. (b) LD50 for normal and Snell cells. (c) Cellular ATP content after doxycycline exposure for 72 hr, and (d) ATP content after 72 hr of exposure to rotenone, which does not induce mitochondrial UPR. Normal cells are represented by blue circles; Snell cells are represented by orange triangles for *N* = 6 mice for each group except that in panel B, the LD50 is shown for 2 independent experiments using *N* = 10 pairs of mice. *indicates *p* < .05 by Student's *t* test for comparison between normal and Snell cells

To see if Snell cells resist lethal and metabolic effects of mitochondrial stress, we measured cell viability using a WST‐1 assay after 72 hr of doxycycline exposure (Figure 3a). Lethal dose 50 (LD50), the dose of doxycycline that kills 50% of the initial population, was twofold higher in Snell cells (Figure 3b). We also measured ATP content after incubating normal and Snell cells in medium containing increasing doses of doxycycline. Normal cells lost 60% of their cellular ATP content after 72 hr of 30 μg/ml doxycycline treatment, whereas Snell cells maintained ATP content under the same conditions. When the doxycycline dose was increased to 60 μg/ml, normal cells were almost devoid of ATP, while Snell cells retained 25% of initial ATP content (Figure 3c).

**Figure 4:**
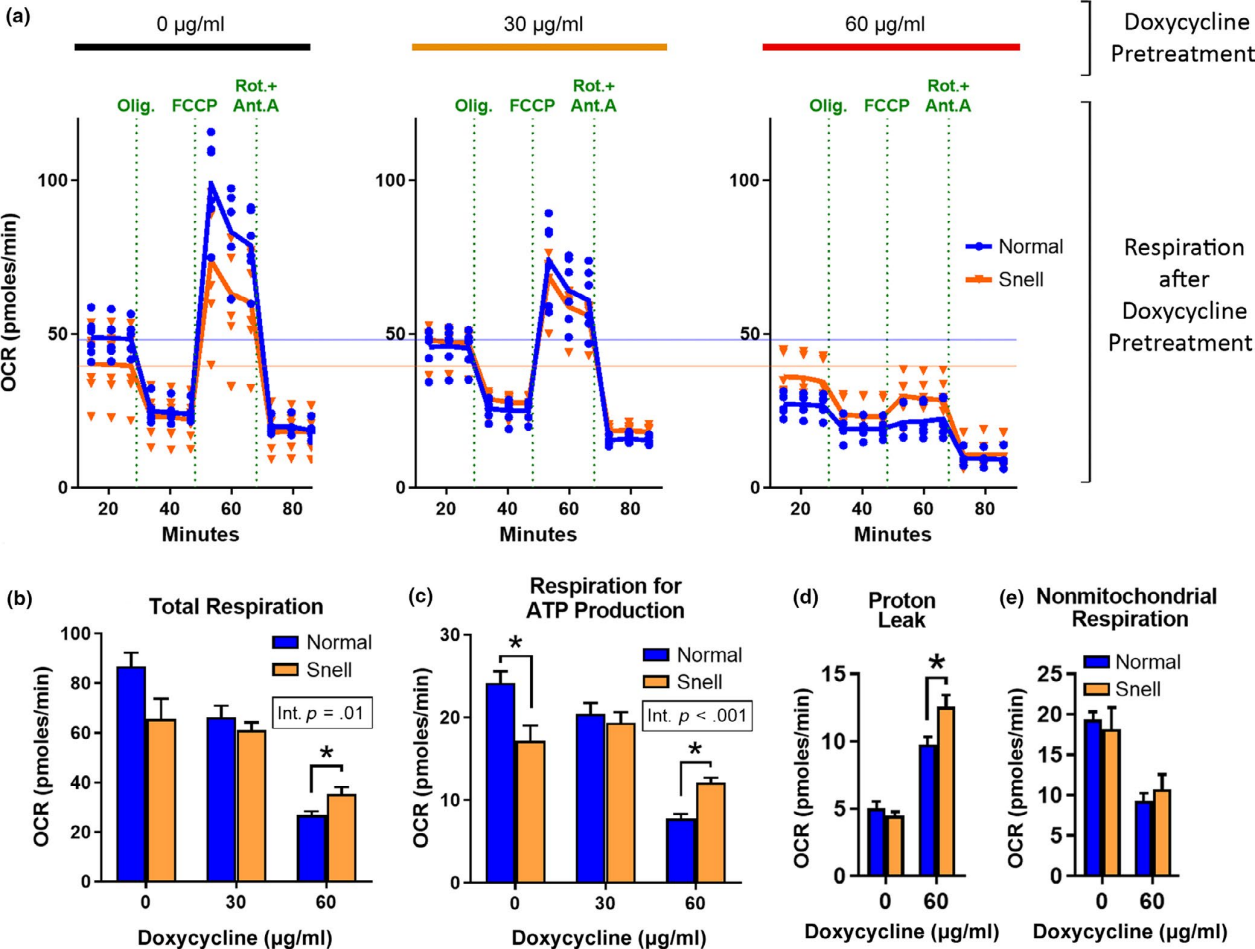
Snell cells maintain mitochondrial function after stress. (a) Respiration curves obtained with or without pretreatment by doxycycline (0, 30, and 60 μg/ml for 24 hr). Each line represents the average OCR for each group of *N* = 6 mice; values for individual mice are shown by blue circles (normal) and orange triangles (Snell). Horizontal blue (normal) and red (Snell) lines are shown across three graphs to serve as reference for basal respiration without prior stress treatment. (b) Total respiration, (c) oxygen consumption linked to ATP production, (d) proton leak, and (e) nonmitochondrial oxygen consumption for *N* = 6 mice. * indicates *p* < .05 by Student's *t* test for comparison between normal and Snell cells

In order to rule out the possibility that preservation of ATP content in Snell cells might be because of a lower ATP consumption rate, we treated cells with rotenone, a chemical inhibitor of ETC complex I, which suppresses ATP production but does not directly disturb mitochondrial proteostasis or induce mtUPR. We reasoned that if Snell cells maintain ATP levels because they consume less ATP, cellular ATP content would also be maintained when ATP synthesis was blocked by rotenone. However, rotenone treatment caused comparable loss of ATP levels in normal and Snell cells, and there was no detectable difference in cellular ATP content (Figure 4d), implying that normal and Snell cells have similar ATP consumption rates under these conditions. Taken together, our results indicate that Snell cells are able to maintain cellular ATP content at a higher level than normal cells after doxycycline‐induced mitochondrial stress.

### 2.4 Snell cells maintain oxidative respiration after doxycycline stress

A more direct indicator of mitochondrial function is the rate of oxidative respiration. It is possible to calculate the rates of total cellular respiration, respiration for ATP production, proton leak, and nonmitochondrial respiration by measuring real‐time cellular oxygen consumption in four phases separated by injections of oligomycin, FCCP, and rotenone with antimycin A, which inhibit different components of the ETC complex (see Methods). In order to assess whether mitochondrial respiration is maintained after pre‐exposure to mitochondrial stress, we exposed primary fibroblasts from normal and Snell mice to doxycycline (0, 30, and 60 μg/ml for 24 hr) pretreatment followed by an hour of rest period in fresh medium in the absence of doxycycline and then measured oxidative phosphorylation rate (Figure 4a). Of note, neither 30 μg/ml nor 60 μg/ml doxycycline treatment induces cell death at this time point, but both trigger mtUPR in primary mouse fibroblasts and hepatocytes (Houtkooper et al., 2013).

At the basal level, where neither Snell nor normal cells had been pretreated with doxycycline, the level of total respiration was similar, with Snell cells showing a slightly lower level that did not differ significantly (*p* = .053) from normal controls (Figure 4b). Further analysis comparing respiration rates before and after complex V (ATPase) inhibition by oligomycin revealed that oxygen consumption utilized for ATP production was 29% lower in Snell cells in the absence of prior doxycycline treatment (Figure 4c). Doxycycline pretreatment, however, lowered both parameters in normal cells, an effect to which Snell cells were resistant: When examined after pre‐exposure to 60 μg/ml doxycycline, Snell cells exceeded normal cells in both basal and oligomycin‐inhibited O_2_ consumption (Figure 4b,c). ATP‐synthesis‐coupled respiration in normal cells pretreated with 60 μg/ml doxycycline decreased approximately 70%, while the loss in respiration rate in Snell cells was only 30%. Thus, when both the normal and Snell cells were pre‐exposed to mitochondrial stress, respiration rates in Snell cells were resistant to the stress effect, and surpassed those in normal cells (Figure 4c).

In order to confirm that the maintenance of respiration rate in Snell cells was not due to inefficient mitochondrial stress induction, we calculated the level of mitochondrial stress‐induced proton leak by comparing the rates of oxygen consumption when only the ATPase (ETC complex V) is blocked by oligomycin to those when the ETC is totally shut down by a combination of rotenone and antimycin A. The level of initial damage induced by doxycycline, measured by proton leak, was higher in doxycycline‐treated Snell cells, demonstrating that the efficacy of stress exposure on Snell cells was at least as high as on normal cells, if not higher (Figure 4d). We observed no significant difference in nonmitochondrial respiration rates (Figure 4e), confirming that higher respiration rate in Snell cells was not due to differences in nonmitochondrial factors such as cell size or cytosolic redox reactions.

### 2.5 *Tfam* and *Pgc1a* expression is constitutively upregulated in Snell mice

**Figure 5:**
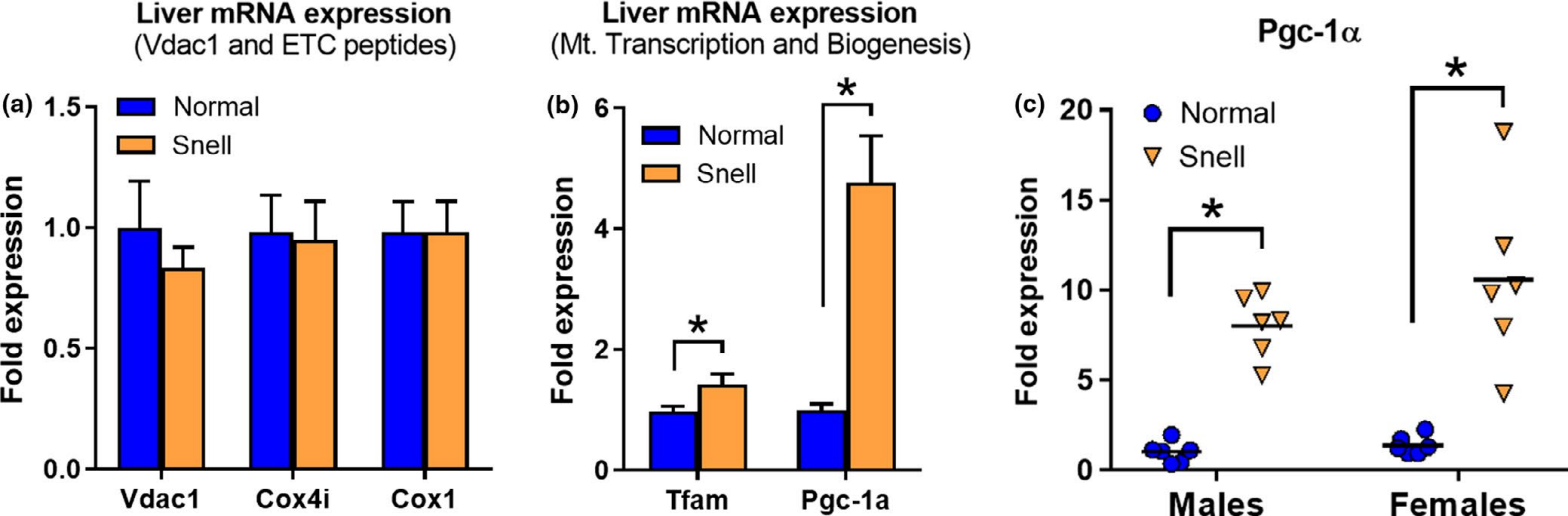
*Tfam* and *Pgc1a* expression is constitutively upregulated in Snell liver. (a) mRNA levels of Vdac1 (also called Porin, which encodes a mitochondrial transport channel protein), Cox4i (nuclear‐DNA‐encoded), Cox1 (mitochondrial‐DNA‐encoded), and (b) Tfam and Pgc‐1α in the liver tissue of normal and Snell mice (*N* = 6, males). (c) Pgc‐1α transcript levels in the liver tissue of an independent set of normal and Snell mice, including both genders (*N* = 6). * indicates *p* < .05 by Student's *t* test for comparison between normal and Snell cells

**Figure 6:**
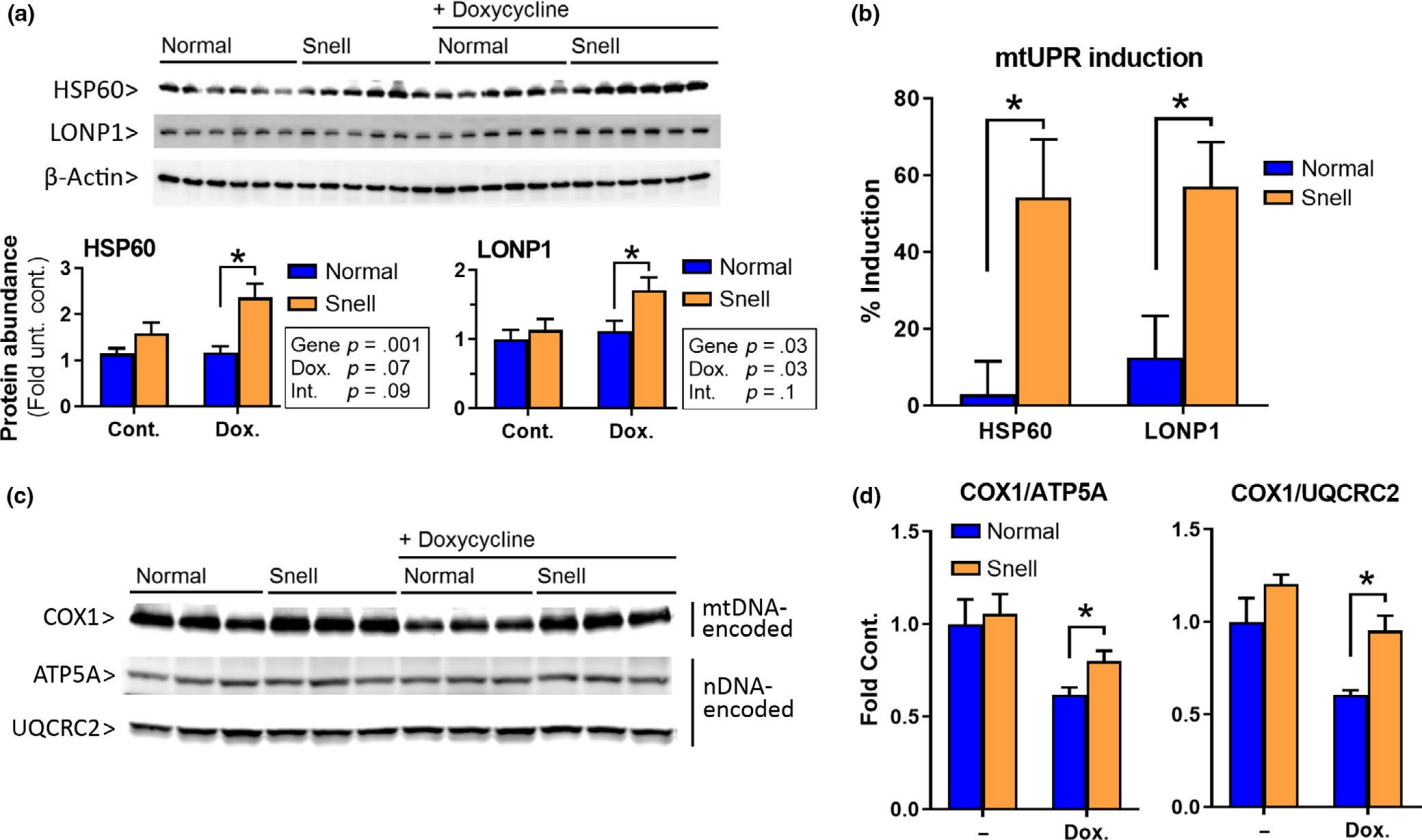
Snell mice show elevated hepatic HSP60 and LONP1 (mtUPR) and can maintain mitochondrial proteostasis after in vivo stress*.* (a) HSP60 and LONP1 protein blots and quantifications, (b) level of HSP60 and LONP1 (mtUPR) induction by doxycycline, (c) mitochondrial‐DNA‐encoded COX1, and nuclear‐DNA‐encoded ATP5A and UQCRC2 protein blots, and (d) mito‐nuclear protein balance measured by COX1/ATP5A and COX1/UQCRC2 ratios for normal and Snell liver samples from mock‐ and doxycycline‐treated mice (*N* = 6). *indicates *p* < .05 by Student's *t* test for comparison between normal and Snell cells

We hypothesized that elements of the mitochondrial biogenesis pathway may have altered regulatory mechanisms in Snell cells, and to test this, we measured *Tfam* and *Pgc1* mRNA expression in liver samples collected from normal and Snell mice. As a control, we monitored mRNA levels of three independently regulated mitochondrial genes in order to detect possible in vivo differences in mitochondrial abundance in normal and Snell livers. *Vdac1* (*Porin*) encodes a mitochondrial transport channel protein which is widely used as mitochondrial loading control. *Cox4i* encodes a nuclear‐DNA‐encoded ETC protein, and *Cox1 encodes* a mitochondrial‐DNA‐encoded ETC protein. We did not find any difference in the mRNA levels of *Vdac1, Cox4i,* or *Cox1* (Figure 5a) or in the levels of mitochondrial proteins ATP5a and UQCRC2 (Figure 6e), suggesting comparable mitochondrial content in the liver of normal and Snell livers. We observed a small but significant elevation in *Tfam* expression (~1.5‐fold, *p* = .03). On the other hand, we detected a dramatic upregulation of *Pgc‐1α* expression (5 ± 2‐fold, *p* < .001) in Snell males (Figure 5b). We further confirmed this result by performing an independent experiment with an additional set of mice comprising both sexes and noted that the phenotype was not gender specific; *Pgc‐1α* expression was also elevated in female Snell mice (Figure 5c). In Snell liver, expressions of TFAM and PGC‐1α are also higher at the protein level (Ozkurede et al., 2019).

### 2.6 Snell mice exhibit improved mitochondrial stress response in vivo

Acute doxycycline treatment of mice has been shown to disturb mitochondrial proteostasis in the liver (Moullan et al., 2015). We exposed normal and Snell mice to in vivo mitochondrial stress by feeding them doxycycline diet for 2 weeks and assessed the level of mtUPR induction and maintenance of mito‐nuclear protein balance as indicators of mitochondrial stress response and protection against mitochondrial stress at the organismal level. We did not detect any effect on body weight in normal or Snell mice. As opposed to our findings in primary fibroblasts, which showed higher basal mtUPR in Snell cells (Figure 1a–d, untreated samples), HSP60 and LONP1 protein levels were comparable in normal and Snell liver samples from mice on the control diet. However, when the mice were exposed to acute mitochondrial stress by in vivo doxycycline treatment, both HSP60 and LONP1 protein levels were higher in Snell liver samples (Figure 6a). Average HSP60 induction in response to doxycycline‐mediated mitochondrial stress was 2.9% in normal mice and 54.3% in Snell mice (*p* = .01). Similarly, average LONP1 induction was 12.6% in normal mice and 57.2% in Snell mice (*p* = .02; Figure 6b).

To determine whether Snell mice were resistant to disruption of mitochondrial proteostasis, we measured levels of COX1 (mitochondrial‐DNA‐encoded), ATP5A (nuclear‐DNA‐encoded), and UQCRC2 (nuclear‐DNA‐encoded) proteins (Figure 5c). Because doxycycline inhibits mitochondrial protein synthesis without affecting cytosolic protein synthesis, we expected to observe suppression of COX1, with no effect on ATP5A and UQCRC2, which would result in an imbalance between mtDNA‐ and nDNA‐encoded proteins in the mitochondria. In the absence of doxycycline treatment, both COX1/ATP5A and COX1/UQCRC2 ratios were comparable in normal and Snell mice (Figure 6d, untreated mice). As predicted, doxycycline treatment inhibited COX1 expression in the liver (Houtkooper et al., 2013), but the disruption was weaker in Snell mice: COX1/ATP5A ratio was 0.62 in normal and 0.80 in Snell livers; COX1/UQCRC2 ratio was 0.61 in normal and 0.95 in Snell livers. Importantly, the weaker disruption observed in Snell mice was not due to lower efficacy of mitochondrial stress treatment since the induced stress response was higher in Snell liver (Figure 6b, Figure S2).

## 3 DISCUSSION

The effects of mitochondria on health and longevity gained attention when unbiased RNAi screens in *C. elegans* showed that at least one‐third of the longevity‐associated genes encode proteins required for mitochondrial function (Hamilton et al., 2005; Lee et al., 2003). Follow‐up studies identified the upregulation of mtUPR as one mechanism linking mitochondrial changes to nematode lifespan extension (Durieux et al., 2011), which was further supported by the observation that overexpression of a single mtUPR gene (*hsp70F*) was sufficient to extend lifespan in *C. elegans* (Yokoyama et al., 2002). Compared to lower organisms, lifespan experiments on mice are more expensive and time‐consuming. Accordingly, only a small percentage of null mutations of interest are tested for their pro‐longevity effects in mice. As a complementary approach, comparison of long‐lived mice to matched controls focusing on specific molecular pathways can provide useful insights into the molecular mechanisms involved in increased mouse longevity. Here, we used Snell dwarf mice to assess whether improved mtUPR is a feature of one of the longest‐lived single‐gene mouse models. Our results indicate improved mtUPR in the liver of Snell mice in response to doxycycline‐induced mitochondrial stress and provide a rationale for future mouse lifespan experiments involving compounds, like doxycycline, that induce mtUPR.

Primary fibroblasts from Snell dwarf mice show higher resistance to cellular stressors such as UV light, heat, paraquat, hydrogen peroxide, and cadmium (Murakami, Salmon, & Miller, 2003). Here, we report that Snell cells are also resistant to mitochondrial stress, specifically the effects of doxycycline on cell viability (Figure 3a,b), cellular ATP content (Figure 3c), and oxidative respiration (Figure 4). Increased mitochondrial stress resistance observed at the cellular level in Snell fibroblasts is consistent with higher mtUPR (HSP60 and LONP1) levels detected (Figure 1a–d). Snell cells also exceeded controls in their ability to maintain *Cox1* (mtDNA‐encoded) transcript levels after doxycycline treatment (Figure 2a). Following up on this phenotype, we found that in response to mitochondrial stress, the mRNA levels of Pgc‐1α, the main regulator of mitochondrial biogenesis, were upregulated exclusively in Snell cells. Snell‐cell‐specific induction of PGC‐1α was also detected at the protein level and was not specific to the agent used to induce mitochondrial stress (Figure 2c,d). In vivo, *Pgc‐1α* mRNA expression in Snell liver was 5‐ to 10‐fold higher than in normal controls (Figure 5b), independent of sex (Figure 5c). Protein levels of both TFAM and PGC‐1 are also upregulated in Snell liver (Ozkurede et al., 2019). Previously, elevated Pgc‐1α expression was reported in the liver and kidney of GHR‐KO mice (Al‐Regaiey, Masternak, Bonkowski, Sun, & Bartke, 2005; Gesing, Bartke, Wang, Karbownik‐Lewinska, & Masternak, 2011), which also have defective GH signaling and show lifespan extension, suggesting that the mitochondrial biogenesis pathway may be regulated by GH/IGF1 signaling. Supporting this idea, mice overexpressing GH show decreased Pgc‐1α expression (Al‐Regaiey et al., 2005). On the other hand, calorie restriction (CR), which induces robust lifespan extension in wild‐type mice with intact GH/IGF1 signaling pathways, also upregulates PGC‐1α protein levels in liver (Al‐Regaiey et al., 2005), suggesting a possible scenario where PGC‐1α‐regulated mitochondrial biogenesis might be important for mammalian longevity independent of GH/IGF1 signaling. Further experiments are required to address whether upregulation of mitochondrial biogenesis contributes to maintenance of health at old age and extended lifespan.

In addition to local mtUPR induction, muscle‐specific mitochondrial stress has been reported to inhibit insulin signaling at the organismal level in *Drosophila* (Owusu‐Ansah, Song, & Perrimon, 2013), suggesting a cell‐nonautonomous effect of mitochondrial stress on IGF1/insulin signaling, at least in the fly. Our findings, indicating an improved mtUPR pathway in Snell mice with deficient in IGF1/GH signaling, also suggest that the proposed interaction may be reciprocal such that subnormal IGF1/GH signaling might potentiate the mtUPR pathway, as well.

It is plausible that the improved mitochondrial stress resistance observed in Snell cells may be a manifestation of the combined effects of mtUPR and mitochondrial biogenesis. The elevated mtUPR in Snell cells may decrease the response time to mitochondrial stress and minimize irreversible protein aggregation, while, in parallel, the mitochondrial pool may be replenished through concurrent upregulation of the mitochondrial biogenesis pathway. Alternatively, Snell cells may have elevated mitochondrial turnover. In support of this hypothesis, the ratio of mitochondrial protein synthesis rate to DNA synthesis rate is higher in Snell heart and muscle (Drake et al., 2015). Since we observe indications of elevated mitochondrial biogenesis in Snell cells without any detectable increase in mitochondrial abundance (Figure 1e–g), the rate of mitochondrial clearance, that is, mitophagy, may be upregulated as well. It is likely that mitophagy plays an active role in restoring mitochondrial homeostasis after mitochondrial stress exposure in Snell cells. Thus, the combined effects of elevated mitophagy and upregulated mitochondrial biogenesis may provide a possible mechanism for maintained mitochondrial function in Snell cells. Further studies are required to test these suggestions.

The first indications of age‐related decline in mitochondrial health were provided by histological studies of human and rat tissue samples fifty years ago (Tauchi & Sato, 1968). Mitochondrial function declines in the liver (Yen, Chen, King, Yeh, & Wei, 1989), heart, skeletal muscle (Short et al., 2005), kidney, and brain (Ojaimi, Masters, Opeskin, McKelvie, & Byrne, 1999) during aging, while mitochondrial stress increases through accumulation of defects such as mtDNA mutations (Corral‐Debrinski et al., 1992; Yen, Su, King, & Wei, 1991), damaged mitochondrial proteins (Bakala et al., 2003), and loss of mitochondrial proteostasis (Benzi et al., 1992). Despite the increase in the mitochondrial stress load during aging, a corresponding increase in the mtUPR has not been reported. In contrast, LONP1 activity in rat liver is 49% lower in old animals (Bakala et al., 2003). In mouse skeletal muscle, *Lonp1* expression decreases with age (Bota, Van Remmen, & Davies, 2002), and these age‐related declines can be totally prevented by caloric restriction at a level that extends rodent lifespan (Lee, Klopp, Weindruch, & Prolla, 1999). Recent evidence suggests that reversal of the age‐related decline in mtUPR may prove beneficial for mammalian health: muscle stem cells from old mice show decreased expression of mtUPR genes *Hsp60* and *Hsp10*, and nicotinamide riboside treatment starting at old age (22–24 months), which reverses this decline in the mtUPR pathway, induces neurogenesis and increases mean lifespan of old mice (Zhang et al., 2016).

Supporting the pro‐longevity role of mtUPR in mammals, we report that Snell dwarf mice show “potentiated” mtUPR; Snell liver exhibits elevated mtUPR induction in response to in vivo mitochondrial stress, despite the absence of such a response in normal mice. Both HSP60 and LONP1 mtUPR proteins are ~50% elevated in Snell livers after 2 weeks of doxycycline treatment, accompanied by a weaker disruption of mito‐nuclear protein balance in these mice (Figure 6c,d). This observation, in principle, suggests that it may be possible to induce a maintained upregulation of mtUPR, a protective pathway, without accommodating stress‐induced molecular defects. Induction of mtUPR in normal mice may require higher or more prolonged doses of doxycycline, or other agents, or may be detectable by a more comprehensive analysis of multiple tissues at varying intervals after stress exposure.

In summary, our results show elevated mtUPR in a long‐lived mouse model, both in live mice and in cultured cells. Interestingly, although both the basal and poststress levels of mtUPR were elevated in primary fibroblasts freshly isolated from Snell mice, at the organismal level, mtUPR levels in Snell liver was higher than those in normal controls *only* after doxycycline‐induced mitochondrial stress exposure (Figure 6a). It would be of interest to evaluate mtUPR in tissues of long‐lived mouse models harboring modifications that could upregulate mtUPR, such as *Surf1^−/−^* mice (Dell'agnello et al., 2007; Pharaoh et al., 2016). Our findings suggest that examination of the mtUPR pathway in other long‐lived mouse models—not only at the basal level, but also after acute in vivo mitochondrial stress exposure—may provide invaluable insights on the involvement of mtUPR in mammalian aging. Once the molecular mechanism behind elevated mtUPR induction seen in Snell mice is uncovered, it may be possible to exploit genetic or pharmaceutical treatments to enhance mitochondrial stress response in aging mammals.

## 4 EXPERIMENTAL PROCEDURES

Snell dwarf and littermate control mice were produced by crossing (DW/J × C3H/HeJ) *Pit1^dw/+^* heterozygous parents. Snell mice with dw/dw genotype were identified by their small size at the age of 3 weeks. Heterozygotes and +/+ mice, which are not distinguishable phenotypically, were used as normal littermate controls. The mice were either euthanized or used in doxycycline treatment experiments at the age of 6 months. All experiments were performed in accordance with guidelines and regulations provided by the University of Michigan University Committee on Use and Care of Animals.

For the in vivo doxycycline treatment experiments, 6‐month‐old male Snell mice and normal siblings (*N* = 6 per group) were fed control or doxycycline chow, corresponding to 50 mg/kgBW/day doxycycline intake. Weight measurements were taken on days 0, 2, 5, 10, and 14 of doxycycline treatment. The mice were dissected on day 15. Liver tissue samples were frozen in liquid nitrogen and stored at −80°C until analysis.

Full Methods are available in the Supporting Information.

## CONFLICT OF INTEREST

The authors have no conflict of interest to declare.

## AUTHOR CONTRIBUTIONS

U.O. and R.M. conceived the experiments and wrote the manuscript. U.O. performed the experiments and analyzed the data.
