# Supplementary figures and images for: Improved mitochondrial stress response in long‐lived Snell dwarf mice
Source: Aging Cell. 2019 Aug 18;18(6):e13030. doi: 10.1111/acel.13030 (PMC6826134; doi:10.1111/acel.13030)

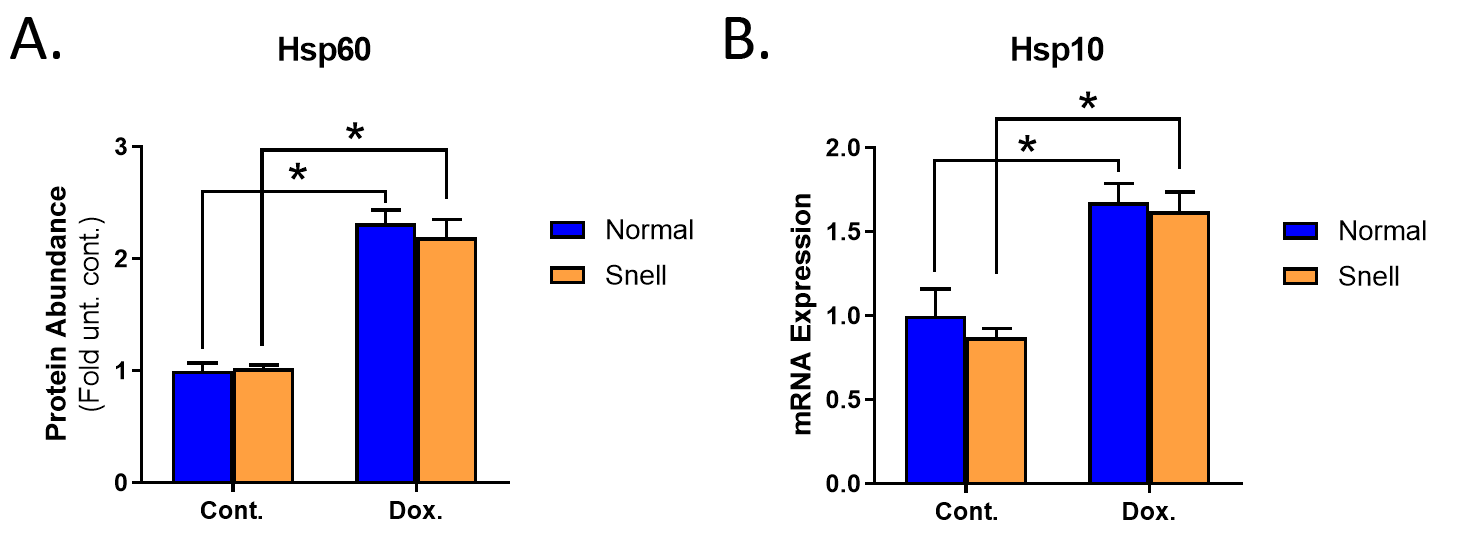

Supplement: Supplementary file 1 [file ACEL-18-e13030-s001.tif]

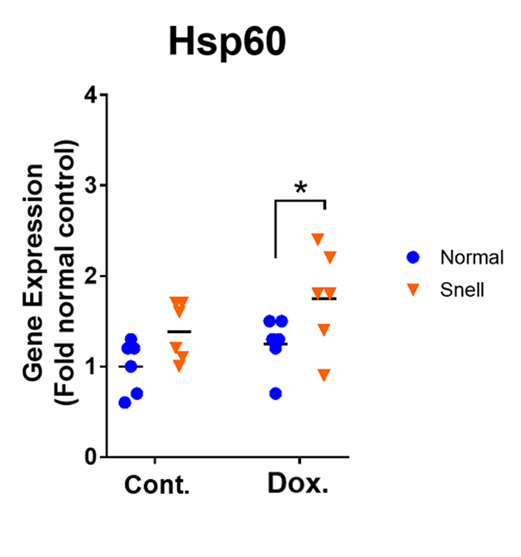

Supplement: Supplementary file 2 [file ACEL-18-e13030-s002.tif]

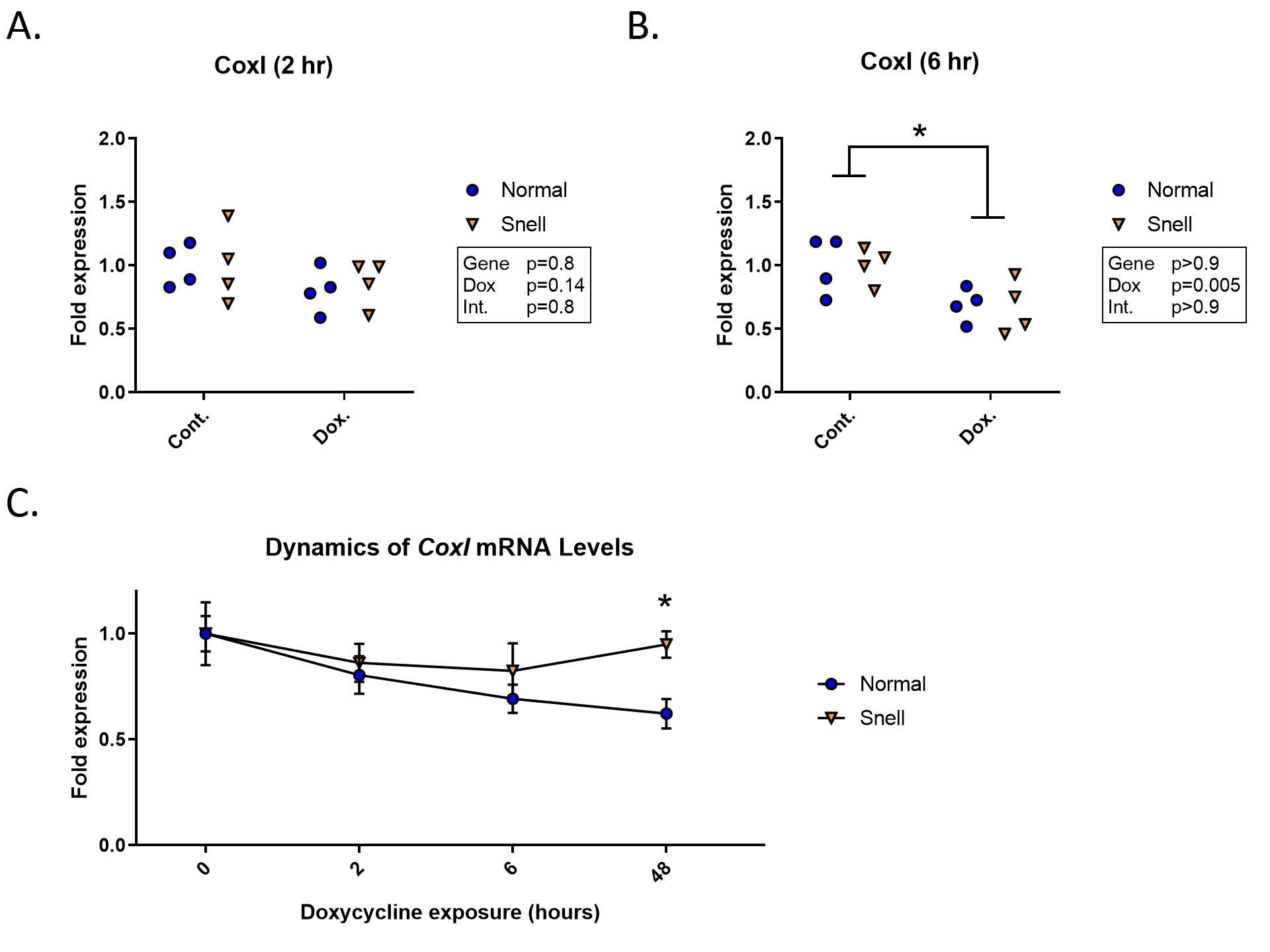

Supplement: Supplementary file 3 [file ACEL-18-e13030-s003.tif]
