# Supplementary material for: Improved mitochondrial stress response in long‐lived Snell dwarf mice
Source: Aging Cell. 2019 Aug 18;18(6):e13030. doi: 10.1111/acel.13030 (PMC6826134; doi:10.1111/acel.13030)
Supplement: Supplementary file 4 [file ACEL-18-e13030-s004.docx]

Supplementary Information for

Improved Mitochondrial Stress Response in Long-lived Snell Dwarf Mice

Ulas Ozkurede and Richard A. Miller

Richard A. Miller

E-mail: millerr@umich.edu

**This PDF file includes:**

Supplementary Text (Full Materials and Methods)

Figures S1-3.

# Supplementary Information Text

## Materials and Methods

### Mice

Snell dwarf and littermate control mice were produced by crossing (DW/J x C3H/HeJ) Pit1^dw/+^ heterozygous parents. Snell mice with dw/dw genotype were identified by their small size at the age of 3 weeks. Heterozygotes and +/+ mice, which are not distinguishable phenotypically, were used as normal littermate controls. The mice were housed in microisolator cages with 1/8” Bed-O-Cob bedding (The Andersons, Maumee, OH). The mice had free access to tap water and Purina 5001 Rodent Chow (St. Louis, MO). Snell mice were caged with normal sized females to prevent premature death from hypothermia. The mice were either euthanized or used in doxycycline treatment experiments at the age of 6 months. All experiments were performed in accordance with guidelines and regulations provided by the University of Michigan University Committee on Use and Care of Animals.

### In-vivo Doxycycline Treatment

6-month-old male Snell mice (N=6) and normal siblings (N=6) were fed control (BioServ, S4207) or doxycycline chow (BioServ, S3888), corresponding to 50 mg/kgBW/day doxycycline intake. Weight measurements were taken on day 0, 2, 5, 10, and 14 of doxycycline treatment. The mice were dissected on day 15. Liver tissue samples were collected and frozen in liquid nitrogen. Frozen tissue samples were reduced to powder using a mortar and pestle, and stored at -80^°^C.

### Primary Fibroblast Cell Culture

Growth media (GM) was prepared by supplementing DMEM (Gibco, 11965-092) with 10% FBS and 1% penicillin/streptomycin (Gibco, 15140-122). Fibroblast isolation media (FIM) was prepared by dissolving Collagenase Type II (Gibco, 17101-015) in GM (400U/ml), and sterilized by passing through a 0.2 μm filter. Tail snips (~5 mm) were taken from 6-month-old Snell mice and normal siblings, washed with ethanol twice, and rinsed in PBS. Individual tail snips were transferred into 60 mm petri dishes containing a few drops of FIM, and minced using sterile scalpels to produce pieces smaller than 1 mm on each side. 4 ml of FIM was added to each dish, and minced tails were incubated 24 hours at 37^o^C, 10% CO_2_. After incubation, the media with minced tail pieces was pipetted up and down several times, passed through sterile nylon netting (100 μm) to remove larger pieces, and centrifuged at 1000 g for 3 minutes. Supernatant was removed. The cells were re-suspended in 10 ml GM, seeded on 10 cm cell culture dishes and incubated at 37^o^C, 10% CO_2_. When confluent, the cells were transferred to 15 cm dishes. After no more than 3 weeks of growth, primary fibroblasts were used for experiments.

### Mitochondrial Stress Treatments

The cells were counted and seeded on cell culture dishes (10^6^ cells in 10 ml media per 10 cm dish) in GM. After 6 hours, GM was removed and replaced with cell media (CM; serum-free DMEM with no antibiotic). The next day, CM was removed and replaced with fresh CM containing either 30 μg/ml doxycycline (Santa Cruz, sc204734), 50 μg/ml thiamphenicol (Millipore Sigma, T0261), or an equal volume of diluent as control. The cells were incubated at 37^o^C, 10% CO_2_ for time intervals indicated for each experiment. For treatments longer than 24 hours, the medium was replaced every day with fresh CM containing the compound of interest. At the end of treatment, the medium was removed. Cells were carefully washed with PBS, scraped, collected in 1 ml cold PBS, transferred to microcentrifuge tubes, and centrifuged for 3 minutes at 1000 g at 4°C to remove PBS. Cell pellets were used for protein or RNA extraction.

### Measurement of Cell Viability, LD50, and Cellular ATP Content

For cell viability assays, cells were seeded on 96-well cell culture plates (30 x 10^3^ cells in 100 μl medium per well) in GM. After a 6-hour incubation, GM was replaced by CM. The next day, the medium was removed and CM containing doxycycline at the indicated concentrations was added. After 72 hours, WST-1 Cell Proliferation Reagent (Millipore Sigma, 05015944001) was added (10 μl/well), and the plate was incubated for 4 hours. Absorbance at λ = 440 nm was measured using a microplate spectrophotometer. The LD50 (Lethal Dose 50), the doxycycline dose corresponding to 50% decrease in formazan dye signal compared to untreated controls, was calculated.

For ATP content measurements, the cells were seeded (30 x 10^3^ cells in 100 μl media per well) on opaque-walled 96-well cell culture plates in order to prevent luminescence cross talk between wells. After 6-hour incubation GM was replaced with CM. The next day, the medium was removed and CM containing doxycycline or rotenone (Millipore Sigma, R8875) at the indicated concentrations was added. After 72-hour incubation, ATP content was measured using CellTiter-Glo® Assay Kit (Promega, G7571) as described in the product protocol. Luminescence signal was measured on a SynergyTM HT Multi-Mode Microplate Reader (BioTek® Instruments, Inc). All incubations were performed at 37^o^C, 10% CO_2_.

### Measurement of Real-time Oxidative Consumption

Real time measurements of oxygen consumption rates were performed using the XF Cell Mito Stress Test Kit (Agilent Technologies, 103015-100), XF FluxPak mini cartridge pack (Agilent Technologies, 102601-100), and XF Base medium (Agilent Technologies, 102353-100). Cells were seeded (104 cells in 200 μl GM per well) on a 96 well XF cell culture plate. After 6 hours, GM was replaced with CM and the cells were incubated overnight. The next day CM was removed and CM containing 0, 30, or 60 μg/ml doxycycline was added to induce mitochondrial stress. The cells were left to incubate for 24 hours at 37oC, 10% CO2. In parallel, the sensor cartridge was placed in a utility plate (Agilent Technologies, 102601-100) containing 200 μl/well XF Calibrant (Agilent Technologies, 102601-100) and incubated overnight at 37oC in non-CO2 incubator for hydration. The next day, assay medium (AM) containing 1 mM pyruvate, 2 mM glutamine, and 10 mM glucose in XF Base medium was freshly prepared, warmed to 37oC, adjusted with 0.1 N NaOH to have pH of 7.4, and sterilized by filtering. The cells were examined for confluence and washed with AM by discarding 175 μl media and adding 175 μl AM three times, in order to prevent cell disruption. After the wash, 175 μl media was removed and 150 μl AM was added, leaving 175 μl AM in each well. The cells were left for incubation at 37oC in non-CO2 incubator for 45 minutes. In parallel, Oligomycin (8 μΜ), FCCP (9 μΜ), and Rotenone/Antimycin A (5 μΜ) solutions were prepared in AM and loaded to cartridge ports A, B, and C respectively (25 μl per port). Then the cartridge with the utility plate was placed in a Seahorse XFe96 Extracellular Flux Analyzer (Agilent Technologies), and calibration was started. At the end of 45-minute incubation, the utility plate in the XFe96 Extracellular Flux Analyzer was replaced with the cell plate and the measurement protocol was started. The operating software Wave (Agilent Technologies) was programmed in advance to perform measurements every ~6 minutes, injecting 25 μl oligomycin solution (final 1 μΜ) at t=28 min, 25 μl FCCP solution (final 1 μΜ) at t=47 min, and 25 μl Rotenone/Antimycin A solution (final 0.5 μM) at t=66 minutes. Oxygen consumption rate at each time point was calculated by Wave software according to readings collected from the XFe96 Extracellular Flux Analyzer. Each data point is the average of 5 technical replicates.

### Calculation of Respiration Parameters

Respiration parameters were calculated by analyzing real-time oxygen consumption rate (OCR) measurements in four phases separated by injections of oligomycin, FCCP, and rotenone/antimycin A, which inhibit or activate specific pathways allowing the dissection of individual components of respiration. Total respiration is the oxygen consumption rate measured before the addition of oligomycin (OCRt=27). Respiration for ATP production was calculated by subtracting the oxygen consumption rate after inhibition of ATP synthase (ETC Complex V) by oligomycin, which is the oxygen consumption that is not linked to ATP production, from total respiration rate (OCRt=27 - OCRt=46). Non-mitochondrial respiration is the reading after the mitochondrial ETC is totally blocked by addition of Complex I/III inhibitors Rotenone and Antimycin A (OCRt=85). Total mitochondrial respiration is calculated by subtracting non-mitochondrial respiration from total cellular respiration (OCRt=27 - OCRt=85). Proton leak was calculated by subtracting respiration for ATP production from total mitochondrial respiration ([OCRt=27 - OCRt=85] – [OCRt=27 - OCRt=46] = OCRt=46 - OCRt=85).

### Western Blot and Antibodies

Cell pellets and tissue samples (reduced to powder) were lysed in 200 μl RIPA buffer with protease inhibitor cocktail (ThermoFisher, 78430) by incubating on ice for 20 minutes, vortexing every five minutes for 5 seconds each time. The lysates were centrifuged for 20 minutes at 8000 g at 4°C. The supernatants were collected in new tubes. Protein concentration was estimated using BCA Protein Assay (ThermoFisher, 23225) and adjusted accordingly. For tissue samples, where protein yield was high, the lysates were diluted 1:10 in lysis buffer. After addition of Laemmli protein sample buffer containing β-mercaptoethanol (1.42 M), each lysate was divided into two tubes: lysates in the first group of tubes were boiled for 5 minutes at 100°C before electrophoresis, while the lysates in the second group were incubated for 15 minutes at 37°C. The latter were used for analysis of mitochondrial proteins including COXI, which is highly hydrophobic and becomes insoluble upon denaturation by boiling. Samples were run on polyacrylamide gel and transferred to PVDF membranes. The membranes were blocked with 2% BSA in TBST solution (20mM Tris, 500 mM sodium chloride, 0.9 mM polyoxyethylene-20-sorbitan monolauraten deionized water), and probed with primary antibodies β-Actin[C4]-HRP (Santa Cruz, sc47778HRP), PGC-1α (Millipore, ST1202) , HSP60 (Santa Cruz, sc1052), or LONP1 (Abcam, ab103809) at 1:1000 dilution for incubation on a shaker for 18 hours at 4°C. Mitochondrial ETC proteins COX1, ATP5a, and UQCRC2 were detected using MitoProfile® Total OXPHOS Rodent WB Antibody Cocktail (Abcam, ab110413). Mouse IgG-HRP (Santa Cruz, sc2031), Goat IgG-HRP (Santa Cruz, sc2350), and Rabbit IgG-HRP (Cell Signaling, 7074S) were used as secondary antibodies at 1:2000 dilution for incubation on a shaker for 90 minutes at room temperature. To minimize non-specific binding, the membranes were washed three times in TBST for 10 minutes each time after primary and secondary antibody incubations. Protein bands were detected using enhanced chemiluminescent substrate (ThermoFisher, 34075) and a chemiluminescence imager. Signal strength was quantified using ImageJ software.

### mRNA Extraction and q-rtPCR

Frozen tissue samples (reduced to powder) or cell pellets were lysed in 1 ml TRIzol reagent (ThermoFisher) by sonication on ice for 30 seconds with 1 second on/off cycles. The lysates were transferred to pre-cooled Phase Lock Gel-Heavy tubes. Chloroform (0.2 ml for 1 ml TRIzol) was added. After 3 minutes of incubation at room temperature, the tubes were centrifuged for 15 minutes at 7500 x g at 4°C. Aqueous phase (~0.5 ml) on the top was transferred to new tubes. Isopropanol (0.5 ml) was added. After 10-minute incubation at room temperature, the RNA was precipitated by centrifugation at 7500 x g at 4C. The supernatant was discarded. The precipitated RNA was washed with 75% ethanol (1 ml), and then centrifuged for 5 minutes at 7500 x g at 4°C. The supernatant was carefully discarded. Precipitated RNA was air-dried and resuspended in 50 μl RNase-free water. RNA concentration was measured by nanodrop. cDNA was produced by iScript cDNA Synthesis Kit (BioRad, 1708890) according to protocol provided. qPCR was performed using Fast SYBR^TM^ Green Master Mix (ThermoFisher, 4385612) according to product protocol. The following primer pairs were used [Gene, PubMed Gene ID, forward primer sequence, reverse primer sequence]: Hsp60, 15528, tgtttggagaagaggggttg, cgctcgttcagcttttcttt; Hsp10, 15528, gggtcaggagggaaaggaaa, cagcttcacgtgacaccatt; Lonp1, 74142, tggttgagctcctgagaagg, aacttgtctccgaggtcctg; Vdac1, 22333, agaggtacagcagaaacccc, aggtgtgtacatgcttccga; Ppargc1a, 19017, atgtgtcgccttcttgctct, atctactgcctggggacctt; Tfam, 21780, agccaggtccagctcactaa, aaacccaagaaagcatgtgg; mt-Co1, 17708, tcatcccttgacatcgtgct, gtctgagtagcgtcgtggta; Cox4i (Cox4i1), 12857, ccatgtcacgatgctgtctg, ctcccaaatcagaacgagcg.

### Statistical Analysis

Prism (GraphPad Software, Inc) was used for statistical analysis and graphical representation of the data. Error bars represent SEM. Comparison between groups of two were performed using Student’s t-test. For experiments involving more than one variable, two-way ANOVA was used to analyze individual effects of and interaction between the two factors. Post-hoc (Tukey) analysis was used following 2-way-ANOVA to calculate p values for differences between doxycycline-exposed normal and Snell samples. Differences with p values smaller than 0.05 were considered significant.


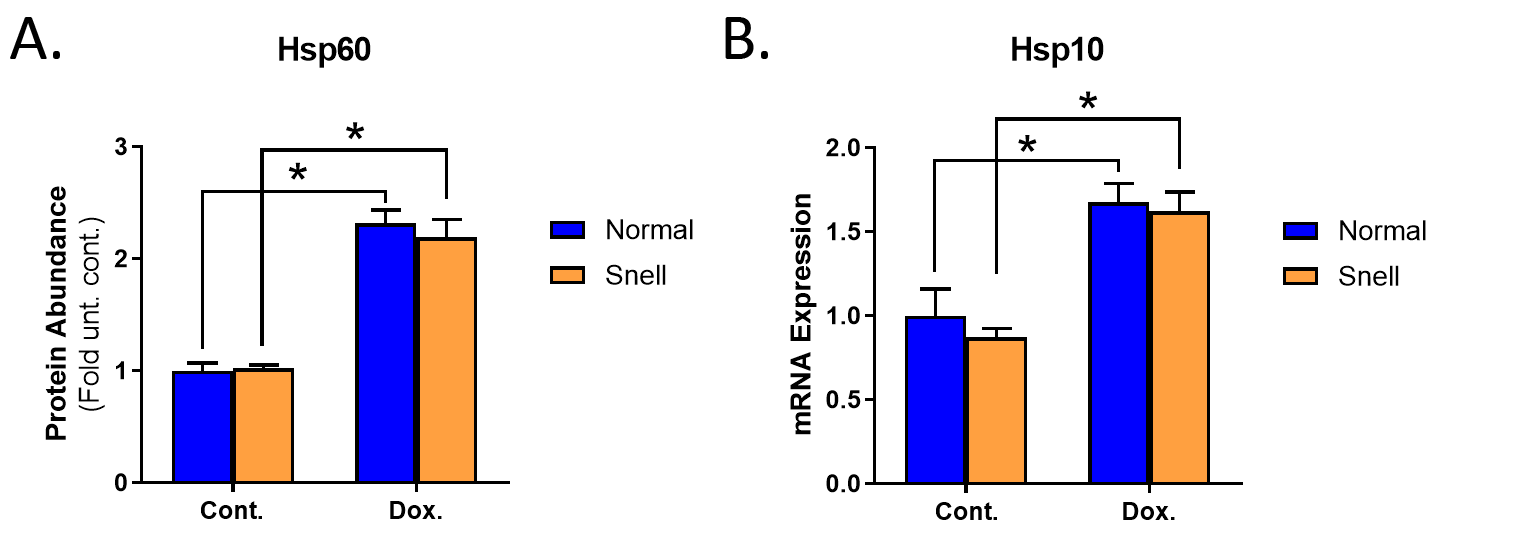


## Figure S1. Snell cells respond to doxycycline treatment.

(A) Transcript levels of mtUPR genes *Hsp60* and (B) *Hsp10* in untreated and doxycycline-treated primary fibroblasts from normal and Snell mice (N=6). (*) indicates p < 0.05 by Student’s t-test for comparisons between Cont. and Dox. samples for each genotype.

##
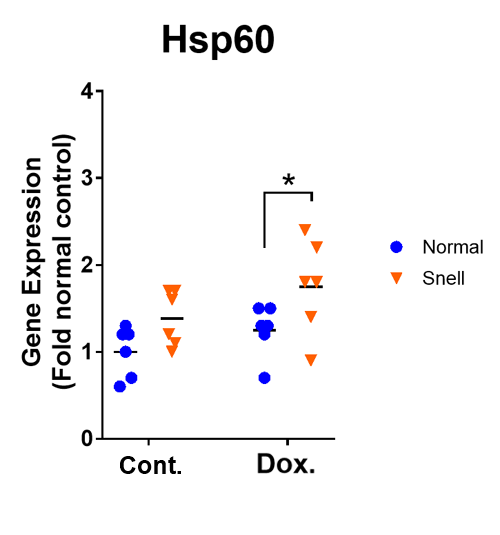


## Figure S2. Hsp60 is induced at the mRNA level in Snell livers.

*Hsp60* mRNA levels in liver tissue samples from normal and Snell mice after exposure to acute mitochondrial stress by in-vivo doxycycline treatment. Each circle represents a normal mouse; each triangle represents a Snell mouse (N=6 mice per group). (*) indicates p < 0.05 by Student’s t-test for comparison between normal and Snell samples.


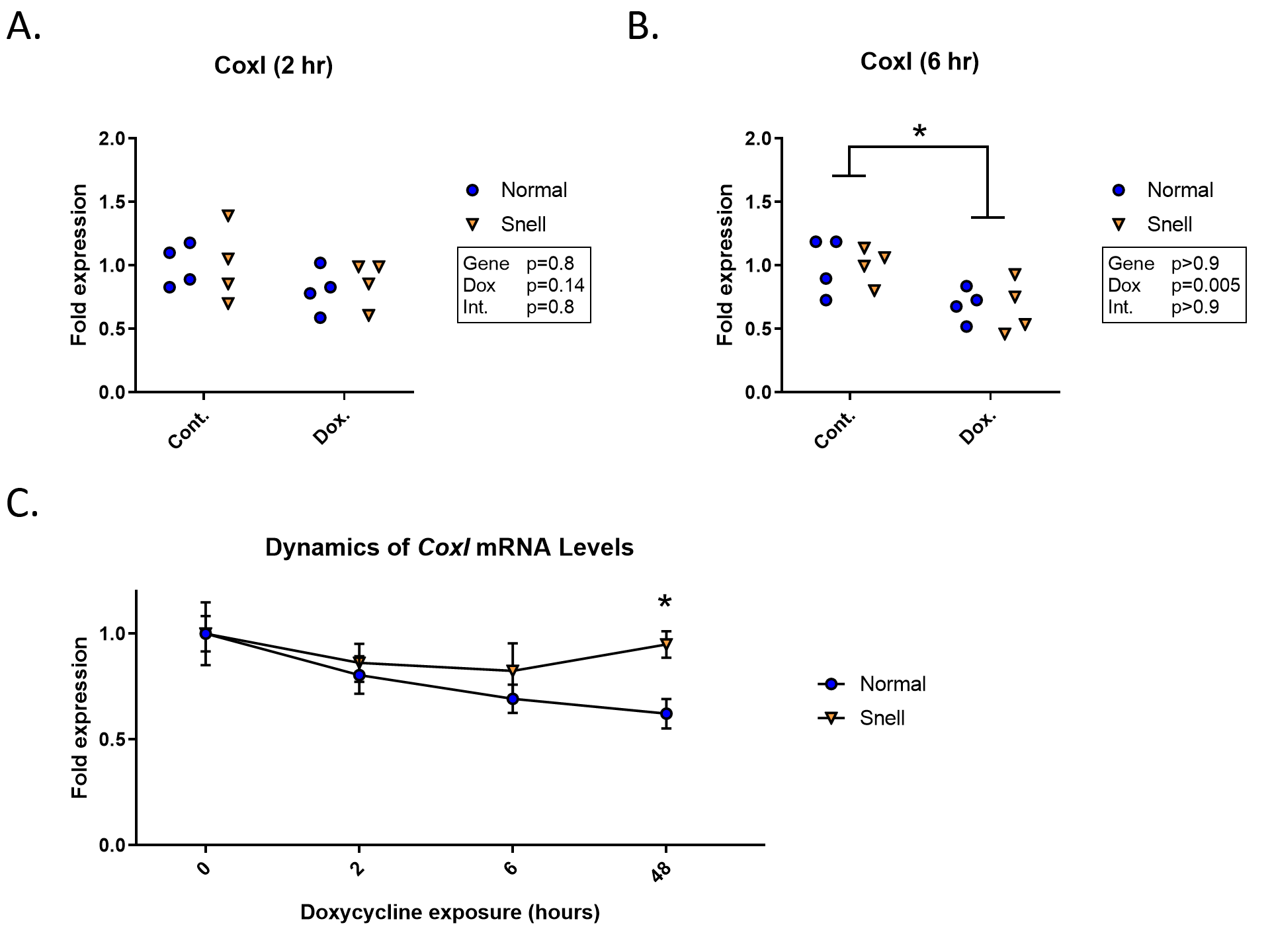


## Figure S3. Snell cells recover from doxycyline-induced decay in *CoxI* expression by 48 hours.

Mitochondrial-DNA-encoded *CoxI* mRNA levels in primary fibroblasts after doxycyline (30 μg/ml) exposure for (A) 2 hours and (B) 6 hours. Each circle represents cells from a normal mouse; each triangle from a Snell mouse (N=4 mice per group). In panel B, (*) indicates significant doxycycline effect independent of the genotype. (C) *CoxI* mRNA levels after 2, 6, and 48 hours of doxycycline exposure. Each data point represents average of samples from normal (circles) or Snell (triangles) mice (N=4-10 mice per group). Error bars show SEM. (*) represents significant treatment-genotype interaction effect by 2-way-ANOVA analysis.
